# Coronavirus OC43 and Influenza H3N2 Concomitant Unilateral Parotitis: The Importance of Laboratory Tests in Mumps-Like Parotitis

**DOI:** 10.3390/pathogens12111309

**Published:** 2023-11-02

**Authors:** Serena Spampinato, Piero Pavone, Giovanni Cacciaguerra, Salvatore Cocuzza, Emmanuele Venanzi Rullo, Silvia Marino, Andrea Marino, Giuseppe Nunnari

**Affiliations:** 1 Department of Clinical and Experimental Medicine, University of Messina, 98124 Messina, Italy; emmanuele.venanzirullo@unime.it; 2 Unit of Infectious Diseases, Department of Clinical and Experimental Medicine, University of Catania, ARNAS Garibaldi Hospital, 95122 Catania, Italy; andreamarino9103@gmail.com (A.M.); giuseppe.nunnari1@unict.it (G.N.); 3 Section of Pediatrics and Child Neuropsychiatry, Department of Clinical and Experimental Medicine, University of Catania, 95124 Catania, Italy; gio.cacciaguerra@gmail.com (G.C.); silvia_marino86@hotmail.it (S.M.); 4 Department of Medical and Surgical Sciences and Advanced Technologies “GF Ingrassia”, ENT Section, University of Catania, 95124 Catania, Italy; s.cocuzza@unict.it

**Keywords:** parotitis, mumps like, Coronavirus OC43, influenza H3N2

## Abstract

Mumps is an acute generalized infection caused by a *Paramyxovirus*. Infection occurs mainly in school-aged children and adolescents and the most prominent clinical manifestation is nonsuppurative swelling and tenderness of the salivary glands, unilaterally or bilaterally. Negative serology for mumps requires a differential diagnosis with other infectious agents, but it is not routine. An 11-year-old girl presented with fever and right-sided parotitis and a negative serology for Mumps. A respiratory panel revealed the presence of Coronavirus OC43 and influenza virus H3N2. Parotitis may be caused by the parainfluenza virus, Epstein–Barr virus, influenza virus, rhinovirus, adenovirus, or other viruses in addition to noninfectious causes such as drugs, immunologic diseases, or obstruction of the salivary tract as predisposing factors. In this case, Coronavirus OC43 and influenza virus H3N2 were detected. The H3N2 has been already reported in the literature, whereas Coronavirus OC43 has never been associated with parotitis before; although, in the present case, the association of the two viruses does not let us conclude which of the two was responsible for the disease.

## 1. Introduction

Mumps is the most common cause of parotitis, characterized by swelling and tenderness of the salivary glands, unilaterally or bilaterally [1,2].

Mumps-negative cases can be due to a plethora of viruses and bacteria, but they are often left undiagnosed because differential diagnosis with other infectious agents is not routine [3,4,5,6,7,8].

Viruses such as the parainfluenza virus (PIV), Epstein–Barr (EBV), influenza virus (InV), Rhinovirus, Adenovirus, and other viruses have been reported to cause parotitis [1,2,3,4,5,6,7,8].

Since the beginning of the SARS-CoV-2 pandemic, the use of protective face masks and several lockdowns have altered the circulation of many respiratory viruses and the development of humoral and cellular immunity against them in the population, especially in childhood, i.e., influenza [3,9].

In the case report, we describe the case of an 11-year-old girl with unilateral parotitis associated with simultaneous infection of two viruses: the influenza virus H3N2 and the Coronavirus OC43. We underline the importance of making a differential diagnosis through appropriate laboratory tests that take into consideration the most probable causes of parotitis.

## 2. Case Report

In November 2022, an otherwise healthy 11-year-old girl presented with a day of malaise and a fever of up to 38 °C. She was up to date with her immunizations, including the measles–mumps–rubella vaccine. At presentation, the girl was hemodynamically stable with a completely normal physical examination. A combined oropharyngeal and nasopharyngeal swab was negative for SARS-CoV-2 by reverse transcription–polymerase chain reaction. She has been treated with acetaminophen for the fever.

**Figure 1:**
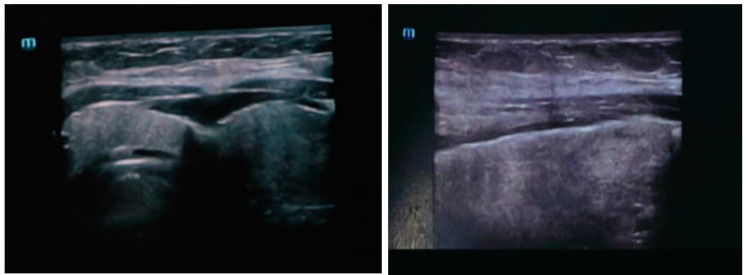
The ultrasound examination showed right parotid gland and right submandibular gland enlarged inhomogeneous echo structure with diffused increase in echogenicity of the glandular parenchyma. There was also a diffuse increase in intraparenchymal vascularity and reactive adenitis. No fluid collection, obstructing stones, dilatation of salivary duct, masses, or abscesses were observed.

Ten days later, however, the child was re-admitted to this institution with right-sided facial swelling, sialadenitis, pain, and persistent fever. Clinical examination was normal, heart rate 85 beats per minute, blood pressure 115/74 mmHg with peripheral oxygen saturations of 98% on room air. A chest radiograph was normal. The ultrasound examination showed the right parotid gland and right submandibular gland enlarged in the homogeneous echo structure with the diffused increase in echogenicity of the glandular parenchyma. There was also a diffuse increase in intraparenchymal vascularity and reactive adenitis. No fluid collection, obstructing stones, dilatation of salivary duct, masses, or abscesses were observed [Figure 1]. We observed clear saliva spontaneously discharging. No pus was excreted from the parotid glands.

Blood laboratory test results showed a total white cell count of 4.9 × 10^6^/μL and a neutrophil count of 4.9 × 10^6^/μL. C reactive protein was slightly elevated [Table 1]. Liver and kidney function tests were normal, as was the sweat test for cystic fibrosis. Thyroid function was normal. Serum amylase was 83 U/L (normal 28–100). Serology was positive (IgG) for measles and rubella, but negative for mumps (IgG and IgM), in spite of vaccination. Anti SARS-CoV-2 IgG were positive [Table 1]. Hepatitis A, hepatitis C, Cytomegalovirus, Epstein–Barr, parvovirus, human immunodeficiency virus IgM, and IgG were negative.

A respiratory panel (BIOFIRE, FILMARRAY Respiratory Panel, BioMérieux, Marcy-l’Étoile, France) was positive for influenza H3N2 and coronavirus OC43, but negative for Adenovirus, Coronavirus NL63, HKU1 and 229E, Adenovirus, Human Metapneumovirus, Human Rhino/enterovirus, Influenza B, Parainfluenza 1,2,3,4 Respiratory Syncitial Virus, MERS-Cov, SARS-CoV-2, Bordetella pertussis, Bordetella parapertussis, Chlamydophila pnumoniae, Mycoplasma pnumoniae. A gastrointestinal panel test was negative (FILMARRAY™ Gastrointestinal, Panel tests, BioMérieux) [Table 2].

The patient continued treatment with non-steroidal anti-inflammatory drugs until parotitis resolved 13 days later. Serology for mumps (IgG and IgM) remained negative.

## 3. Discussion

We report on an 11-year-old girl with an acute onset of unilateral parotitis following three days of fever, with no pre-existing chronic illnesses, and a positive respiratory panel for H3N2 influenza virus and Coronavirus OC43. The decision to conduct a comprehensive viral search panel was prompted not only by the clinical evidence of parotid involvement but especially because of the negative result for the mumps virus. This led us to test for other pathogens that might be implicated in such symptomatology.

This infection primarily manifests in school-aged children and adolescents, with the prominent clinical feature being non-suppurative swelling and pain of the salivary glands, often involving one or both parotid glands. Additionally, fever, headache, muscle pains, and asthenia are characteristic [1]. The involvement of the parotid glands can either be an initial symptom or a consequence of the action of other pathogens [1,2].

Purulent bacterial parotitis may also occur, and it is characterized by the sudden onset of unilateral solid erythematous swelling in the preauricular area above the parotid gland. Occasionally, the swelling may extend to the corner of the jaw. This is associated with local pain, and tenderness [3]. The bacteria that are the most frequent cause of suppurative parotitis include Staphylococcus Aureus, oral aerobes, anaerobes, and Haemophilus Influenzae. In Institution acquired infections, and in immunocompromised patients, methicillin-resistant S. Aureus (MRSA), Enterobacteriales, and Pseudomonas Aeruginosa can also be found. Parotitis can also be polymicrobial [4,5].

Of importance, mumps-like illness can be caused by PiV, EBV, InV, Rhinovirus, Adenovirus, and others [8,9,10,11,12].

As of the current literature, cases of parotitis caused by Coronavirus OC43 have not been reported. Within the Coronavirus family, the infection caused by SARS-CoV-2 associated with parotitis is the most extensively documented in the literature because. SARS-CoV-2, being a multi-faceted disease, the spectrum of symptoms extends well beyond respiratory issues. Many otolaryngologists have observed an increase in the number of patients with acute parotitis potentially linked to COVID-19. Some studies support the hypothesis that intraparotid lymphadenitis is a contributing factor [8].

The potential direct spread of SARS-CoV-2 to the tissues of the parotid gland could theoretically occur due to the presence of angiotensin-converting enzyme 2 in the parotid tissue. The potential risk might be linked to the excretion of virions through saliva [8]. The oral cavity, often considered a reservoir for pathogens, could employ a similar mechanism for the dissemination of other viruses, such as the Coronavirus OC43, detected in the proband.

In the present case, the respiratory panel analysis also revealed the presence of H3N2 influenza virus. Influenza-associated parotitis appears to predominantly coincide with H3N2 influenza virus infection, as reported in numerous studies on sporadic cases of parotitis [9].

During the 2014–2015 influenza season in the United States, 256 cases of influenza-associated parotitis were recorded. A case–control study and a laboratory investigation were conducted to further explore this rare manifestation of influenza. In this study, painful unilateral parotitis was observed in 68% of the described cases [9]. Furthermore, as early as the 2009–2011 period, sporadic cases of mumps were investigated in the United States, gathering epidemiological information, serum samples, oral swabs, and oropharyngeal swabs [10].

In another study conducted from 2007 to 2011 in Barcelona, samples from patients negative for mumps virus but presenting with unilateral or bilateral parotitis were analyzed [11]. They were tested for EBV using real-time PCR, and for respiratory viruses using two multiplex PCR-based tests to detect PIV, InV A, B, and C, Respiratory Syncytial Virus, Enterovirus, Coronavirus 229E, Coronavirus OC43, and Rhinovirus. Out of 101 oral samples, 53 were collected on the first day of glandular swelling, and 74 within the first two days. Viruses were detected in 52 of the samples. One virus was detected in 44 patients, two viruses in 7 patients, and three viruses in 1 patient. It was found that 72.3%, or 73 out of 101 suspected cases of sporadic parotitis reported, were not actually cases of mumps.

The timing of oral sample collection is crucial for interpreting, accurately, negative results for mumps, especially when suspected cases occur in vaccinated individuals [11].

In our study, a negative mumps-like condition was found for the mumps virus, but it tested positive for two viruses: Coronavirus OC43 and Influenza A H3N2 virus. While there is substantial evidence for the occurrence of parotitis due to the sole presence of Influenza A H3N2 virus [9,13,14], the same cannot be asserted for Coronavirus OC43. The literature does not report cases where Coronavirus OC43 acts alone as a triggering factor for parotitis. The question to consider is whether Coronavirus OC43 is not a cause of parotitis because it is unable to initiate the infection on its own, or simply if it is not tested in all cases of parotitis not caused by the mumps virus.

Up to 30% of mumps virus infections can be asymptomatic or present with nonspecific respiratory symptoms, making the recognition and clinical diagnosis of mumps complex [1,2]. Laboratory methods used to confirm mumps include detecting Immunoglobulin M (IgM) antibodies against the mumps virus, demonstrating a specific antibody response to the virus (such as at least a fourfold increase in Immunoglobulin G [IgG] titers measured through quantitative assays, or seroconversion from negative to positive using a standard serological test with matched acute and convalescent serum samples), detecting mumps virus RNA through reverse transcription polymerase chain reaction (RT-PCR) in either conventional or real-time methods, or isolating the mumps virus in cell cultures [10].

As a matter of fact, in a study by Barskey et al. on mumps-like illness, out of 101 case samples analyzed the pathogen has been identified in just 38 cases. Of note, their samples were not tested for either Influenza or Coronaviruses [10].

In light of the various epidemiological and clinical considerations, it is advisable to conduct comprehensive screening tests in cases of Mumps-like disease. This is because cases with a negative laboratory result for mumps are generally classified as suspected cases of parotitis, and the differential diagnosis with other infectious agents is not customary. Furthermore, taking into account the evidence regarding the control of mumps and influenza infections [9], and considering the present case, it is advisable to consider influenza in the differential diagnosis among patients with acute parotitis during the influenza season, especially because a pharmacological treatment is available for it.

## 4. Conclusions

In the present report, Coronavirus OC43 and influenza virus H3N2 were detected along the course of acute unilateral parotitis. Mumps-negative parotitis can be due to several pathogens that need to be thoroughly investigated.

## Figures and Tables

**Table 1:** Laboratory tests and serology for common pathogens.

Laboratory Tests	Results	Normal Values
RBC	4.96 × 10^6^/μL	3.7–5.40 × 10^6^/μL
HgB	14.8 g/dL	11.5–15.5 g/dL
WBC	3.75 × 10^3^/uL	5.2–12.40 × 10^3^/μL
PLT	239 × 10^3^/μL	150–400 × 10^3^/μL
C Reactive Protein	5.3 mg/L	0–5 mg/L
Parvovirus IgG	<0.1	>1.1 Positive
Parvovirus IgM	0.24	>1.1 Positive
Mumps IgG	<5 AU/mL	≥11 Positive
Mumps IgM	0.2 AU/mL	≥1.1 Positivo
Epstein–Barr VCA IgM	<10 U/mL	>40 Positive
Epstein–Barr VCA IgG	<5 U/mL	>40 Positive
Measles Ig G	231 AU/mL	>16.5 Positive
Anti-Rubella virus IgG	28.8 UI/mL	>10 Positive
Cytomegalovirus IgG	0 AU/mL	>6 Positive
Cytomegalovirus IgM	0.30	1.0 Positive

RBC: Red blood cells; HgB: Hemoglobin; WBC: White blood cells; PLT: Platelets.

**Table 2:** Respiratory panel (BIOFIRE, FILMARRAY Respiratory Panel, BioMérieux) for main viruses and bacteria.

PATHOGEN	NUCLEIC ACID TEST
Adenovirus	NUCLEIC ACID NOT DETECTED
Coronavirus 229E	NUCLEIC ACID NOT DETECTED
Coronavirus HKU1	NUCLEIC ACID NOT DETECTED
Coronavirus NL63	NUCLEIC ACID NOT DETECTED
Coronavirus OC43	**NUCLEIC ACID DETECTED**
Human Metapneumovirus	NUCLEIC ACID NOT DETECTED
Human Rhinovirus/Enterovirus	NUCLEIC ACID NOT DETECTED
Influenza A	**NUCLEIC ACID DETECTED**
Influenza B	NUCLEIC ACID NOT DETECTED
Parainfluenza Virus 1	NUCLEIC ACID NOT DETECTED
Parainfluenza Virus 2	NUCLEIC ACID NOT DETECTED
Parainfluenza Virus 3	NUCLEIC ACID NOT DETECTED
Parainfluenza Virus 4	NUCLEIC ACID NOT DETECTED
Respiratory Syncytial Virus	NUCLEIC ACID NOT DETECTED
MERS-CoV	NUCLEIC ACID NOT DETECTED
SARS-CoV-2	NUCLEIC ACID NOT DETECTED
Bordetella pertussis	NUCLEIC ACID NOT DETECTED
Bordetella parapertussis	NUCLEIC ACID NOT DETECTED
Chlamydophila pneumoniae	NUCLEIC ACID NOT DETECTED
Mycoplasma pneumoniae	NUCLEIC ACID NOT DETECTED
Adenovirus	NUCLEIC ACID NOT DETECTED

MERS-CoV: Middle East respiratory syndrome Coronavirus; SARS-CoV-2: Severe acute respiratory syndrome Coronavirus 2.

## Data Availability

The data presented in this study are available on request from the corresponding author.
